# It does belong together: cross-modal correspondences influence cross-modal integration during perceptual learning

**DOI:** 10.3389/fpsyg.2015.00358

**Published:** 2015-04-09

**Authors:** Lionel Brunel, Paulo F. Carvalho, Robert L. Goldstone

**Affiliations:** ^1^Laboratoire Epsylon, Department of Psychology, Université Paul-Valéry Montpellier IIIMontpellier, France; ^2^Department of Psychological and Brain Sciences, Indiana University, BloomingtonIN, USA

**Keywords:** brightness–lightness, pitch, cross-modal integration, cross-modal correspondence, perceptual learning

## Abstract

Experiencing a stimulus in one sensory modality is often associated with an experience in another sensory modality. For instance, seeing a lemon might produce a sensation of sourness. This might indicate some kind of cross-modal correspondence between vision and gustation. The aim of the current study was to explore whether such cross-modal correspondences influence cross-modal integration during perceptual learning. To that end, we conducted two experiments. Using a speeded classification task, Experiment 1 established a cross-modal correspondence between visual lightness and the frequency of an auditory tone. Using a short-term priming procedure, Experiment 2 showed that manipulation of such cross-modal correspondences led to the creation of a crossmodal unit regardless of the nature of the correspondence (i.e., congruent, Experiment 2a or incongruent, Experiment 2b). However, a comparison of priming effects sizes suggested that cross-modal correspondences modulate cross-modal integration during learning, leading to new learned units that have different stability over time. We discuss the implications of our results for the relation between cross-modal correspondence and perceptual learning in the context of a Bayesian explanation of cross-modal correspondences.

## Introduction

Perception allows us to interact with and learn from our environment. It allows us to transform internal or external inputs into representations that we can later on recognize, and it also lets us make connections between situations that we have encountered (see Goldstone et al., 2013). In other words, perception can be envisaged as an interface between a cognitive agent and its environment. However, our environment is complex and instable. Processing a situation may require integrating information from all of our senses as well as background contextual knowledge in order to reduce the complexity and the instability of the situation. In that case, what we call a “conscious experience” of a situation should involve an integration of both a particular state of the cognitive system generated by the current situation (i.e., perceptual state) and former cognitive states (i.e., memory state). Accordingly, integration should be a relevant mechanism for both perceptual and memory processes (see Brunel et al., 2009). In this article, cross-modal perceptual phenomena (e.g., cross-modal correspondence) are employed as an effective way to further investigate this integration mechanism and its connection with perception and memory processes.

It is now well established that cross-modal situations influence cognitive processing. For instance, people are generally better at identifying (e.g., MacLeod and Summerfield, 1990), detecting, (e.g., Stein and Meredith, 1993), categorizing (e.g., Chen and Spence, 2010), and recognizing (Molholm et al., 2002) multisensory events compared to unisensory ones. This multisensory advantage takes place regardless of whether the sensory signals are redundant or not (see Teder-Sälejärvi et al., 2005; Laurienti et al., 2006). More interestingly, it also seems that people spontaneously associate sensory components from different modalities together in a particular, fairly consistent, way. For instance, the large majority of people agree that “Bouba” refers to a rounded shape while “Kiki” refers to an angular one (Ramachandran and Hubbard, 2001). Evidence like this shows a non-arbitrary relation between a shape and a word sound (i.e., a cross-modal correspondence, see Spence, 2011). These correspondences between sensory modalities have a direct influence on online cognitive activity.

Cross-modal correspondences modulate performance in cognitive tasks. For instance, in a speeded classification task^1^, participants are faster at identifying the size of a stimulus when it is accompanied by a congruent tone (e.g., a small circle presented with a high-pitched tone; Gallace and Spence, 2006; Evans and Treisman, 2010) rather than an incongruent tone. Similarly, in a temporal order judgment^2^ task, participants perceive congruent asynchronous stimuli (e.g., a small circle presented with a high-pitched tone) as more synchronous than incongruent stimuli (e.g., a large circle presented with a high-pitched tone; Parise and Spence, 2009). In both examples, a particular relation is defined as congruent when the features share the same directional value (e.g., large size and low-pitched sound) and incongruent when the opposite mapping is used (e.g., small size and high-pitched sound). Directional value is a psychologically salient quality because many perceptual dimensions fall on a continuum with psychologically smaller and larger ends (Smith and Sera, 1992). Larger, louder, and lower pitched values are all perceived as having greater magnitudes than their opposing smaller, quieter, and higher pitched values. Using both speeded and non-speeded measures, this magnitude-based congruency effect has been observed between apparently highly distinct features, such as brightness/lightness and pitch (Marks, 1987, see also Marks, 2004), size and pitch (Gallace and Spence, 2006; Evans and Treisman, 2010), and spatial position and pitch (Evans and Treisman, 2010).

The existence of cross-modal correspondences contributes to our understanding of perceptual processes. Historically, perception has been conceived as a modularized set of systems relatively independent of each another (e.g., Fodor, 1983). However, the existence of a correspondence (within or between sensory modalities) indicates that perceptual components are integrated during perceptual processing. Indeed, Parise and Spence (2009) propose that correspondences affect cross-modal integration directly. Thus, congruent stimuli form a stronger integration than incongruent ones and, as a consequence, produce a more robust impression of synchrony. In other words, the perception of a cross-modal object requires not only multiple activations in sensory areas but also the synchronization and integration of these activations. In that case, features sharing the same directional value produce a stronger coupling between the different unimodal sensory signals and are therefore more robustly integrated together (see also, Evans and Treisman, 2010).

Does the fact that cross-modal integration is stronger with features sharing the same directional value mean that cross-modal integration should not be observed with other relations between features? An impressive amount of behavioral (Brunel et al., 2009, 2010, 2013; Zmigrod and Hommel, 2010, 2011, 2013; Rey et al., 2014, 2015) and brain imagery (see Calvert et al., 1997; Giard and Peronnet, 1999; King and Calvert, 2001; Teder-Sälejärvi et al., 2002, 2005) studies provide evidence of cross-modal integration between unrelated features. For instance, Brunel et al. (2009, 2010, 2013) showed that exposing participants to an association between two perceptual features (e.g., a square and a white-noise sound) results in these features being integrated within a single memory trace (or event, see Zmigrod and Hommel, 2013). Once two features have become integrated, the presence of one feature automatically suggests the presence of the other. In this view, integration is a fundamental mechanism of perceptual learning (see also, unitization; Goldstone, 2000) or contingency learning (see Schmidt et al., 2010; Schmidt and De Houwer, 2012).

If this kind of integration mechanism is involved in perceptual learning and cross-modal correspondences modulate integration, cross-modal correspondences might be expected to modulate cross-modal integration during perceptual learning. In the present work we test this hypothesis across two experiments.

The first experiment was designed in order to test an established cross-modal congruency effect between visual lightness and auditory frequency (see Marks, 1987; Klapetek et al., 2012). To do so, we used a speeded classification task in which participants had to discriminate bimodal stimuli (i.e., audiovisual) according either to the lightness of the visual shape or frequency of the auditory tone. We manipulated the relation between the stimuli’s features so that half of them were congruent (i.e., light-gray + high-pitched tone or dark-gray + low-pitched tone) and the other half was incongruent (i.e., the opposite stimuli mapping). Following Marks (1987), we predicted that, irrespective of the task, we should observe an interaction between visual lightness and auditory frequency. Observing such an interaction would indicate cross-modal correspondence between those two dimensions.

Having established this cross-model correspondence, in the second experiment we test our hypothesis that cross-modal correspondences should modulate cross-modal integration during perceptual learning. To do so, we used a paradigm derived from our previous work on cross-modal integration (see Brunel et al., 2009, 2010, 2013). Our paradigm employs two distinct phases. Participants first implicitly learned that a given shape (e.g., a square) was systematically presented with a sound, while another shape (e.g., a circle) was presented without any sound. Then, participants had to perform a tone-discrimination task according to pitch (i.e., low-pitched or high-pitched) in which each tone (i.e., the auditory target/target-tone) was preceded by one of the geometrical shapes previously seen during the implicit learning phase (i.e., visual prime shape). During learning, we showed (see Brunel et al., 2009, 2010, 2013) that participants integrated the visual shape and the auditory tone within a single memory trace and as a consequence the visual prime shape was abled to influence the processing of the target tone. In order to avoid a conceptual or symbolic interpretation of our priming effect (i.e., “square” = “sound”), a manipulation of the stimulus onset asynchrony (SOA) during the second phase was introduced. Previous studies (see Brunel et al., 2009, 2010) have found a modulation of the priming effect depending the level of SOA. Interference was observed when the SOA between the visual prime and the tone target was shorter than the duration of the sound associated with the shape during the learning phase. In this case, there was a temporal overlap between reactivation induced by the prime and tone processing (see Brunel et al., 2009, 2010). Facilitation was observed when the SOA was equal or longer than the duration of the sound associated with the shape during the learning phase. In this latter case, no temporal overlap occurred between simulation of the learned associated sound and target-tone processing so that target-tone processing took advantage of the auditory preactivation induced by the prime (see Brunel et al., 2009, 2010). This succession of interference followed by facilitation indicates that the shape-sound form a perceptual unit that was integrated during learning (see also Brunel et al., 2009 Experiments 2a,b and 3) otherwise we might have observed only a facilitation irrespective the SOA.

Basically, our second experiment used the same general design. However, we introduced a manipulation of the cross-modal correspondence during learning. In Experiment 2a, participants had to learn bimodal congruent stimuli (i.e., either a dark-gray + low-pitched or light-gray + high-pitched) whereas, in the Experiment 2b, participants had to learn bimodal incongruent stimuli (i.e., either a light-gray + low-pitched or dark-gray + high-pitched). This manipulation of cross-modal correspondences during learning helps us directly test an influence of cross-modal correspondence on cross-modal integration during perceptual learning. The manipulation of the congruency of stimuli might be expected to lead to the creation of perceptual units either more or less stable over time. Experiments 2a,b are crucial to test this idea.

First, if learning cross-modal congruent stimuli is at least equally strong as learning seemingly unrelated cross-modal stimuli, we might expect a replication of our previous findings (see Brunel et al., 2009, 2010) in Experiment 2a. That is to say, we should observe an interference effect for SOAs shorter than the duration of the tone at learning (i.e., slower target discrimination when the prime target relation matches, rather than mismatches, the association seen during learning) and a facilitation for SOAs equal to the duration of the tone at learning (i.e., faster target discrimination when the prime target relation matches rather than mismatches the association seen during learning). This result would indicate that participants learned new perceptual units which integrate both perceptual components. Indeed, if such a unit is not created during learning we would only observe a replication of Experiment 1 results in Experiment 2a. That is to say, we should find an interaction between visual lightness and auditory frequency irrespective the manipulation of the SOA.

Then, with Experiment 2b, we might expect two different possibilities. First, learning incongruent stimuli might disrupt the integration mechanism so that we would not observe the same pattern of results as in Experiment 2a. One could predict no priming effect (either interference or facilitation) if there was no integration between the visual and the auditory components during learning. In that case, one might expect a replication of Experiment 1’s results. Alternatively, learning incongruent stimuli might interfere with the integration mechanism. That is to say, integration might still occur but could be weaker than in Experiment 2a. In that case, one would predict the replication of the pattern of results seen in Experiment 2a, but the priming effect (irrespective of the nature of this effect: interference or facilitation) should be less reliable in Experiment 2b compared to Experiment 2a.

## Experiment 1

### Method

#### Participants

Twenty undergraduate students from Indiana University volunteered to participate in exchange for course credit. Participants’ consent was obtained for all participants in compliance with the IRB of Indiana University. All of the participants reported no corrected or uncorrected hearing impairment. All of the participants had normal or corrected to normal visual acuity.

#### Stimuli and Material

The auditory stimuli, generated using Audacity (Free Software Foundation, Boston), were pure tones with a fundamental frequency of 440 Hz (i.e., low-pitched tone) or 523 Hz (i.e., high-pitched tone). Auditory signals were amplified through Sennheiser (electronic GmbH & Co, Wedemark Wennebostel) headphones with an intensity level of ∼75 Db. The visual stimuli were geometric shapes (a 7 cm square and a circle of 3.66 cm radius) that could be displayed in two different shades of gray (CIE L^∗^a^∗^b^3^ setting value in brackets): dark gray (L: 27.96 a: 0.00, b: 0.00), or light gray (L: 85.26, a: 0.00, b: 0.00). Across the different experimental conditions, the shape could be light or dark and the background was set at mid-gray (L: 56.3, a: 0.00, b: 0.00).

All of the experiments were conducted on a Macintosh microcomputer (iMac, Apple inc., Cupertino, CA, USA). Psyscope software X B57 (Cohen et al., 1993) was used to create and manage the experiment.

##### Procedure

After filling out a written consent form, each participant was tested individually in a darkened room during experimental sessions lasting approximately 45 min. The procedure can be understood as a speeded classification task (see Marks, 1987). On each trial, the participant received a composite stimulus (a particular sound + light combination presented simultaneously for 500 ms), one component of the stimulus was accessory and the other was critical. Depending on the trial, participants had to judge either the lightness (i.e., dark versus light) or the auditory frequency (i.e., low-pitched vs. high-pitched) of the stimulus. At the beginning of each trial, participants received a visual warning signal (presented 1000 ms on the screen) indicating which task they had to perform on the upcoming stimulus.

Participants completed a total of 387 trials divided in three blocks. For each trial, they had to indicate their response by pressing the appropriate response key on a QWERTY keyboard. The stimulus-response mapping was counterbalanced between participants whereas the other combinations between our manipulations were randomly counterbalanced within participants.

### Results and Discussion

The mean correct response latencies (RTs) and mean percentages of correct responses (CRs) were calculated across participants for each experimental condition. RTs that deviated from the mean more or less than 2 SDs were removed (this same cut-off was used throughout all of the experiments and never led to exclusion of more than 3.5% of the data).

Separate repeated measures analyses of variance were performed on latencies RT and CRs with subject as a random variable, and Modality (Visual *vs.* Auditory), Tone Frequency (Low-Pitched vs. High-Pitched), and Lightness (Light vs. Dark) as within-subject variables. For clarity, we report here only the analysis regarding the RTs. The results for CR are comparable to those observed for RTs. There was no evidence of a speed-accuracy trade-off – a significant congruency effect (faster RTs for bimodal congruent than incongruent) was always associated with either a significantly lower error rate for congruent pairs or no statistically significant difference.

#### RT Results

**FIGURE 1:**
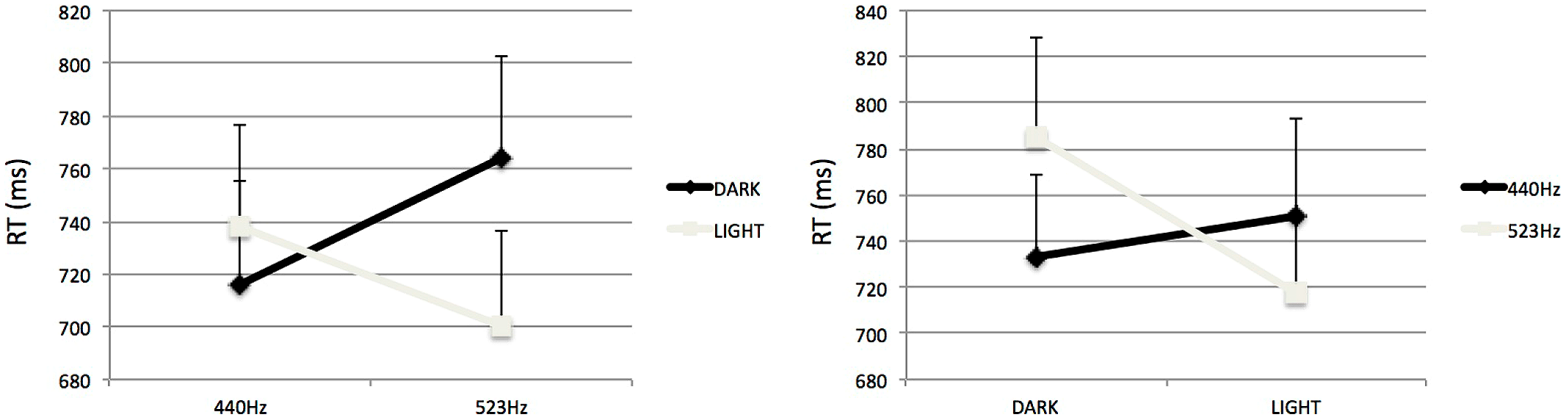
**Mean Reaction times to categorize visual stimuli in Experiment 1, as influenced by frequency of accompanying tone (**left**, visual discrimination task) and to categorize auditory stimuli, as influenced by lightness of accompanying light (**right**, auditory discrimination task).** Errors bars represent ERs of the mean.

As expected, our analysis revealed a reliable significant interaction between the Tone’s Frequency and the Shape’s Lightness, *F*(1,19) = 7.03, *p* < 0.05, $\eta_{p}^{2}$ = 0.27 (see **Figure 1**).

Regardless of the sensory modality of the task, participants were faster to discriminate congruent stimuli (i.e., low-pitched + dark-gray, or high-pitched + light-gray) than incongruent stimuli (i.e., low-pitched + light-gray, or high-pitched + dark-gray). Planned comparisons revealed that participants were faster to categorize low-pitched + dark-Gray stimuli than high-pitched + dark-gray, *F*(1,19) = 8.01, *p* < 0.05. Likewise, participants tended to be faster to categorize high-pitched + light-Gray stimuli than low-pitched + light-gray, *F*(1,19) = 3.55, *p* = 0.07.

We also observed a main effect of Lightness, *F*(1, 19) = 5.09, *p* < 0.05, $\eta_{p}^{2}$ = 0.21. Participants were overall faster to categorize Light-Gray stimuli (mean = 726 ms, SE = 34) than Dark-gray stimuli (mean = 749 ms, SE = 36).

None of the other effects or interactions reached statistical significance.

In this first Experiment, we observed a magnitude-based congruency effect between visual lightness and auditory frequency (see also Marks, 1987). Irrespective of the sensory modality (either visual or auditory), participants were faster to categorize congruent stimuli compared to incongruent stimuli. This is explained by the fact that for the congruent stimuli, the features share the same directional value along the two modalities compared to incongruent stimuli.

Now that we have established a correspondence between lightness and auditory frequency, we can test our prediction that cross-modal correspondence influences cross-modal integration during perceptual learning. This is the aim of Experiments 2a,b.

## Experiment 2a

### Method

#### Participants

Thirty-two undergraduate students from Indiana University volunteered to participate in return for partial course credit. All of the participants reported no corrected or uncorrected hearing impairment. All the participants had normal or corrected to normal visual acuity.

#### Stimuli and Material

We used the same stimuli and materials as in the first experiment. The only difference was that we used four distinct geometrical shapes (namely a square, a circle, and two octagons; see Brunel et al., 2009) equivalent in area. Since participants should categorize visual shapes according to their lightness, we introduced a manipulation of the shapes because variations on a non-relevant dimension has been demonstrated to contribute to improved perceptual learning (Goldstone et al., 2001).

#### Procedure

**FIGURE 2:**
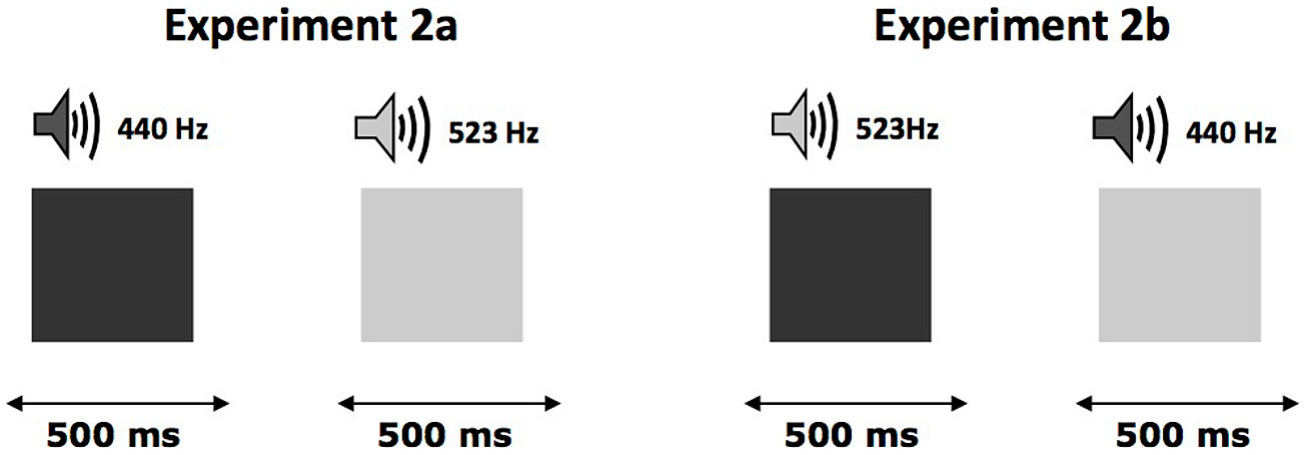
**Organization of the trials in the first phase of Experiments 2a,b.** Each trial consisted in the presentation of a geometric shape displayed in particular level of gray presented simultaneously with a tone. The association between gray-sound and response remained constant during all the learning phase and could be either congruent **(left)** or incongruent **(right)**.

After filling out a written consent form, each participant was tested individually during a session that lasted approximately 15 min. The experiment consisted of two phases. The first phase (learning phase) was based on the hypothesis that the repetition of a sound–brightness association that was not explicitly formulated by the experimenter should lead to the integration of these two components within a single memory trace. Consequently, each trial consisted of the presentation of a shape (either displayed as dark or light gray) for 500 ms. Every shape was presented simultaneously with a tone. Participants were told that their task was to judge, as quickly and accurately as possible, whether the shape was displayed in light or dark gray. They indicated their response by pressing the appropriate key on the keyboard. All of the visual stimuli were presented in the center of the screen, and the intertrial interval was 1,500 ms. For all participants (see **Figure 2**), the shapes displayed in dark gray were presented with the low-pitched tone (440 Hz) and the shapes displayed in light gray were presented with the high-pitched tone (553 Hz). Each gray scale level was presented 32 times in a random order. Half of the participants used their left index finger for the dark-gray response and their right index finger for the light-gray response, while these responses were reversed for the other half of the participants.

**FIGURE 3:**
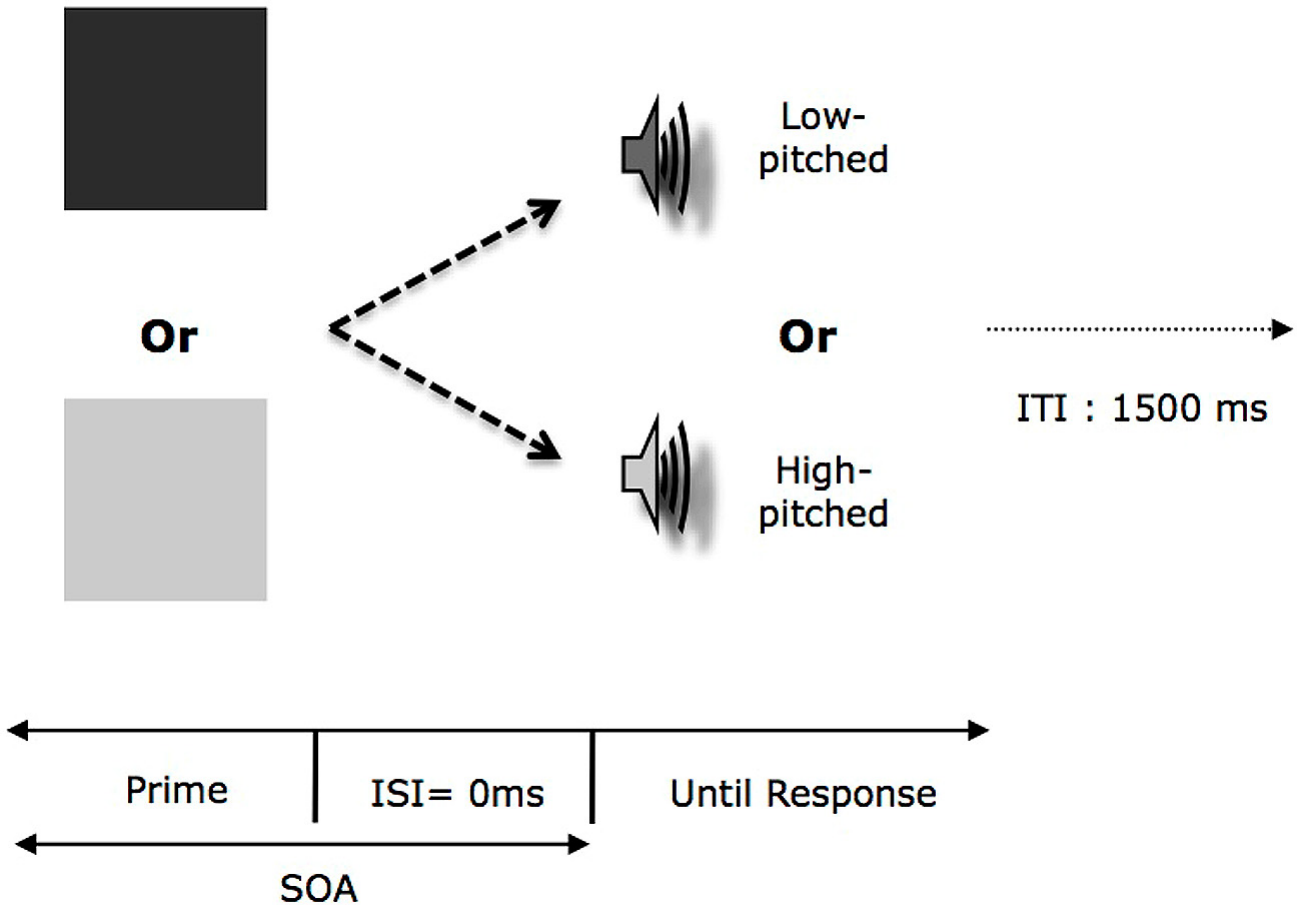
**Organization of one trial in the second phase in Experiments 2a,b as a function of the experimental condition.** Each consisted in the presentation of a visual prime (either displayed in Light or Dark) followed by a target tone. ISI, Interval-Inter-Stimuli; SOA, Stimulus-Onset-Asynchrony; ITI, Interval-Inter-Trial.

The second phase consisted of a categorization task for tones along the pitch dimension (see **Figure 3**). The prime was one shape from the two set of shapes (dark or light gray) presented during the learning phase. In this task, the participants had to judge as quickly and accurately as possible whether the target sound was low-pitched or high-pitched and indicated their choice by pressing the appropriate key on the keyboard. It is important to stress here that all the participants were instructed to keep their eyes open during the entirety of this phase. In order to avoid a conceptual interpretation of our priming effect (i.e., “square” = “sound”), we introduced a manipulation of the SOA (either 100 or 500 ms) during the second phase. We should observe modulation of the priming effect depending on the level of SOA (i.e., an interference for 100 ms SOA followed by a facilitation at 500 ms SOA). Since participants learned specific bimodal congruent stimuli, the relation between prime (i.e., dark prime or light prime) and target (i.e., low or high-pitched tones) could be the same or opposite compared to what was experienced during the learning phase. In addition, for half of the participants the key assignment was the same between the two phases and the opposite for the other half.

Each participant saw a total of 80 trials, 40 with each target sound; half (20) of the target sounds were presented with a shade of gray that had been associated with the corresponding tone during the learning phase, and the other half were presented with a shade of gray that had been associated with the other tone. The order of the different experimental conditions was randomized within and between groups of participants.

### Results and Discussion

#### Learning Phase

The analyses performed on the CRs and on latencies revealed no significant main effects or any interaction. These results are consistent with the idea that participants performed the gray discrimination task accurately (overall accuracy is 93.9%), and the systematic association between a sound and a shade of gray does not impact the visual nature of the task (see Gallace and Spence, 2006 for a similar interpretation). The same patterns of results were found throughout the learning phase in both experiments. This phase led participants to integrate the visual shape and the auditory tone within a single memory trace and as a consequence the visual prime shape should be able to influence the processing of the target tone during the test phase (see also Brunel et al., 2009, 2010, 2013).

#### Test Phase

Separated mixed analyses of variance were performed on latencies (RT) and CRs rates with subject as a random variable, Tone Frequency (Low-Pitched vs. High-Pitched), and Prime-Type (Light vs. Dark) as within-subject variables, and SOA (100 ms vs. 500 ms) as a between-subjects variable.

The analyses performed on the CRs revealed neither a significant main effect (i.e., each *F* < 1) nor any interaction (i.e., each *F* < 1). As far as the RTs were concerned, as expected, our analyses revealed only a significant three-way interaction between SOA, the Tone’s Frequency and the Prime’s Type, *F*(1,30) = 10.16, *p* < 0.05, $\eta_{p}^{2}$ = 0.25. As we can see in **Table 1** the priming effect was reversed for the different SOAs.

**Table 1:** Mean response times (RT) and mean percentages of correct responses (CRs) in each experimental condition in Experiment 2.

		SOA							
	Learning phase	100 ms				500 ms			
		440 Hz		523 Hz		440 Hz		523 Hz	
Prime	Tone	RT (ms)	CR (%)	RT (ms)	CR (%)	RT (ms)	CR (%)	RT (ms)	CR (%)
		**Experiment 2a**							
Dark-Gray	440 Hz	539 (33)	87.6 (3.8)	508 (37)	85.5 (2.4)	504 (45)	82.7(5.0)	540 (46)	82.7 (4.6)
Light-Gray	523 Hz	538 (35)	82.3 (3.8)	558 (39)	83.9 (2.9)	582 (55)	84.1 (4.9)	549 (39)	80.7 (3.3)
Priming Effect		**+1**		**+50**		**-78**		**9**	

		**Experiment 2b**							
Dark-Gray	523 Hz	437 (33)	91.0 (2.3)	470 (36)	84.7 (2.2)	504 (44)	92.5 (1.4)	495 (40)	90.1 (2.4)
Light-Gray	440 Hz	475 (36)	85.2 (2.1)	461 (34)	88.4 (1.7)	477 (39)	90.2 (2.4)	515 (45)	88.8 (2.2)
Priming Effect		**+37**		**+9**		**-27**		**-20**	

SEs in parenthesis. Priming effects were obtained by subtracting the matching condition from the mismatching condition. Negative values indicate facilitation effects whereas positive values indicate interference effects.

**FIGURE 4:**
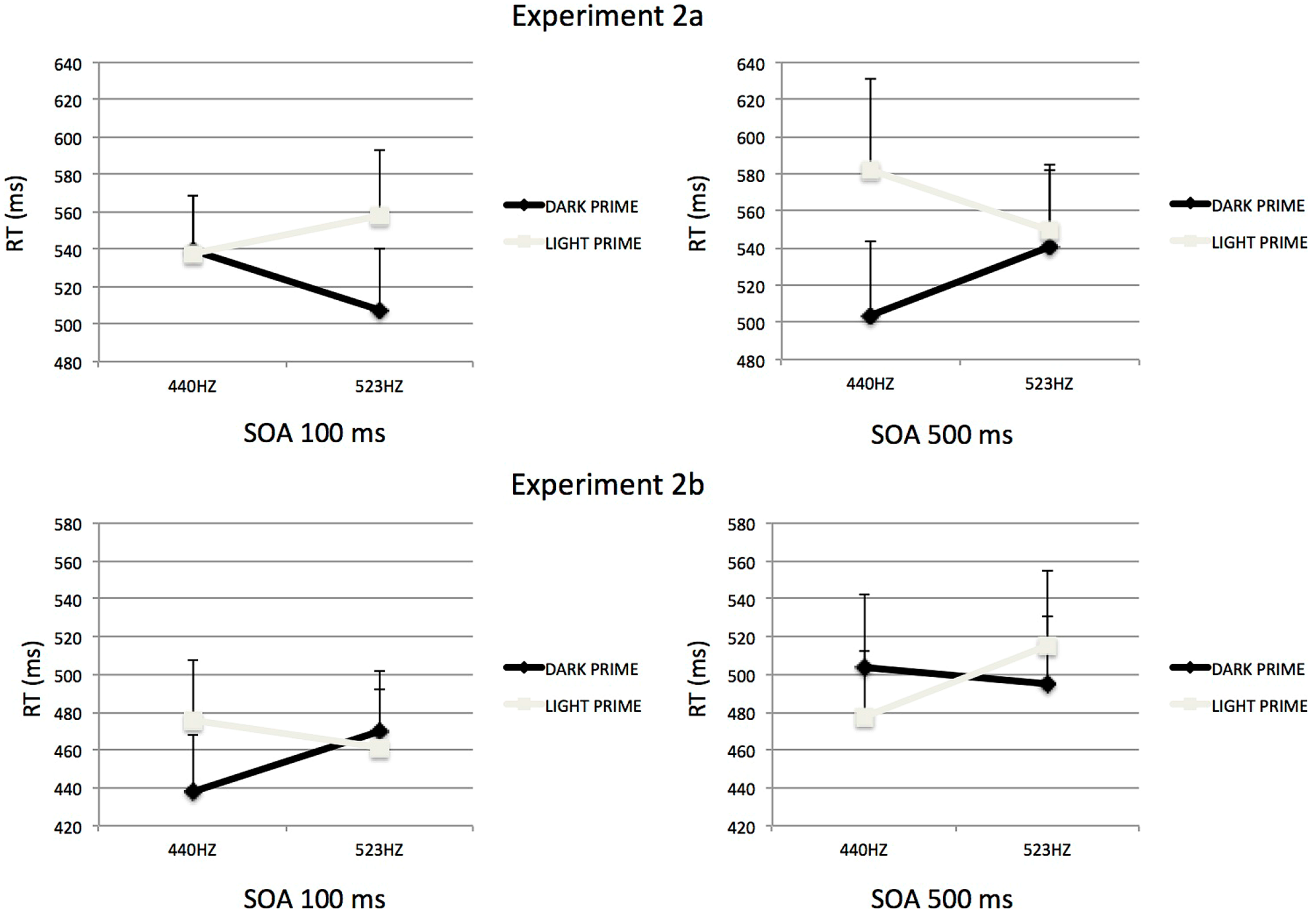
**Mean Reaction times to categorize auditory stimuli, as influenced by visual prime presented 100 ms **(right)** or 500 ms **(left)** in Experiment 2.** The association at learning could be either congruent (**top**; Experiment 2a) or incongruent (**bottom**; Experiment 2b). Errors bars represent ERs of the mean.

Separate analyses of variance were performed for each SOA in order to further investigate these results. For the 100-ms SOA (see **Figure 4**) the analysis revealed a significant interaction between Tone Frequency and Prime-Type,* F*(1,15) = 5.21, *p* < 0.05, $\eta_{p}^{2}$ = 0.26. In that condition of SOA, participants where significantly slower to categorize a high-pitched tone preceded by a light-gray visual prime than a dark-gray visual prime, *F*(1,15) = 11.52, *p* < 0.05. However, for the low-pitched target the type of prime did not influence the categorization, *F* < 1.

For the 500-ms SOA (see **Figure 4**), the analysis revealed a significant interaction between Tone Frequency and Prime-Type,* F*(1,15) = 5.19, *p* < 0.05, $\eta_{p}^{2}$ = 0.26. Participants where significantly faster to categorize low-pitched tones preceded by a dark-gray visual prime than a light-gray visual prime, *F*(1,15) = 15.11, *p* < 0.05. However, for the high-pitched target the type of prime did not influence the categorization, *F* < 1.

The overall pattern of results presented here replicates what was observed in Brunel et al. (2009; Experiment 1). Indeed, we observed an interference effect for 100 ms SOA in which participants were slower at discriminating the target tone when the prime-target relation matched with the association seen during learning compared to when there was a mismatch in the prime-target relation. Conversely, for the 500 ms SOA, we observed that participants were faster at discriminating the target when the prime-target relation matched the association seen during learning. In sum, learning cross-modal congruent stimuli leads to a pattern of results that is comparable with learning cross-modal stimuli that are unrelated. This result indicates that participants have learned new perceptual units which integrate both perceptual components. Indeed, if such a unit were not created during learning we would have only observed a replication of Experiment 1 results in Experiment 2a. That is to say, we should have found an interaction between visual lightness and auditory frequency irrespective of SOA. We turn now to Experiment 2b to explore the role of incongruency in cross-modal correspondence regarding cross-modal integration.

## Experiment 2b

### Method

#### Participants

Thirty-two undergraduate students from Indiana University volunteered to participate in return for partial course credit. All the participants reported no corrected or uncorrected hearing impairment. All of the participants had normal or corrected to the normal visual acuity.

#### Stimuli, Material, and Procedure

We used the same stimuli, materials, and experimental design as in Experiment 2a. The only exception was that participants were exposed to incongruent stimuli (see **Figure 2**) during learning.

### Results and Discussion

#### Test Phase

Separated mixed analyses of variance were performed on latencies RT and CRs rates with subject as a random variable, Tone Frequency (Low-Pitched vs. High-Pitched), and Prime-Type (Light vs. Dark) as within-subject variables, and SOA (100 ms vs. 500 ms) as between-subjects variables. The analyses performed revealed only a significant three-way interaction between SOA, Tone Frequency and Prime-Type, respectively, *F*(1,30) = 14.96, *p* < 0.05, $\eta_{p}^{2}$ = 0.33 for RTs and *F*(1, 30) = 4.83, *p* < 0.05, $\eta_{p}^{2}$ = 0.14 for CR rates. For clarity, we further report here only the analysis regarding the RTs since the results on CR are comparable to those observed for RTs (see **Table 1**). As we can see in **Table 1** the priming effect was reversed for the different SOAs but the same for the different experiments.

Separate analyses of variance were performed for each SOA in order to interpret these results. For the 100-ms SOA (see **Figure 4**) the analysis revealed a significant interaction between Tone Frequency and Prime-Type,* F*(1,15) = 7.15, *p* < 0.05, $\eta_{p}^{2}$ = 0.32. With this short SOA, participants where significantly slower to categorize low-pitched tone preceded by a light-gray visual prime than a dark-gray visual prime, *F*(1,15) = 6.74, *p* < 0.05. However, for the high-pitched target the type of prime did not significantly influence the categorization, *F*(1,15) = 1.07, *p* = 0.31, but the trend is consistent with an interference priming effect, i.e., participants were slower to categorize high-pitched tone preceded by a dark-gray visual prime than a light-gray visual prime (see **Table 1**).

For the 500-ms SOA (see **Figure 4**), the analysis only revealed a significant interaction between Tone Frequency and Prime-Type,* F*(1,15) = 7.84, *p* < 0.05, $\eta_{p}^{2}$ = 0.34. In that condition of SOA, participants where significantly faster to categorize a low-pitched tone preceded by a light-gray visual prime than a dark-gray visual prime, *F*(1,15) = 6.74, *p* < 0.05. However, for the high-pitched target the type of prime did not significantly influence the categorization, *F*(1,15) = 2.84, *p* = 0.11, but the trend is also consistent with a facilitation priming effect, i.e., participants were faster to categorize high-pitched tone preceded by a dark-gray visual prime than a light-gray visual prime.

The overall pattern of results replicates those observed in Experiment 2a. However, the manipulation of the cross-modal correspondence at learning had a significant influence on the size of the priming effect irrespective of interference or facilitation (Mann–Whitney *U* test, *Z* = 1.81, *p* < 0.05), with a smaller priming effect seen for Experiment 2b (Mean = 20 ms) than for Experiment 2a (Mean = 34 ms). This difference might indicate that the decay of the priming effect over time is faster for incongruent stimuli at learning than for congruent stimuli at learning.

## General Discussion

The aim of the present study was to provide evidence in support of the assumption that cross-modal correspondences modulate cross-modal integration during perceptual learning. In our first Experiment, we established a cross-modal correspondence between visual lightness and auditory frequency (see also Marks, 1987). Indeed, regardless of the sensory modality of the task, participants were faster to categorize congruent stimuli (i.e., High-pitched + Light-Gray or Low-pitched + Dark-gray) compared to incongruent stimuli (i.e., the opposite mapping between lightness and auditory frequency). This result is consistent with previous results on cross-modal correspondence (see Gallace and Spence, 2006; Evans and Treisman, 2010; for a review see Spence, 2011). Our second Experiment explored whether learning bimodal congruent or incongruent stimuli influenced the integration mechanism. This idea is consistent with experimental evidence showing that cross-modal integration is involved during perceptual learning (see Brunel et al., 2009, 2010, 2013; Zmigrod and Hommel, 2013) and with experimental work showing that cross-modal correspondences modulate cross-modal integration (see Parise and Spence, 2009). Our experiment used an original priming paradigm that we have designed to study cross-modal perceptual learning (see Brunel et al., 2009, 2010, 2013). In this paradigm, during a learning phase, participants implicitly learn an audiovisual perceptual unit (e.g. a “sound square”). Then, the consecutive phase allows us to test for the existence of such a unit as well as its nature. To do so, we ask participants to categorize target tones preceded by a visual prime. In our previous studies, we showed a priming effect from the visual prime to the target tone limited to the visual prime that was presented with a sound during the learning phase. This result indicates that participants integrated the visual and auditory features and thus the presence of one feature as a prime automatically triggers the other. In the same vein, Meyer et al. (2007) showed that processing of a visual component (i.e., a red flash) that was previously presented with a sound (i.e., a telephone ringing) produced auditory cortex activation. Most interestingly, the manipulation of the SOA during the test phase allows us to rule out a conceptual or symbolic interpretation of the priming effect. In our previous studies, depending on the SOA value (i.e., shorter or at the same duration than the duration of the association during learning), we observed either an interference effect or a facilitation effect. The facilitation or interference of the priming effects depends on the temporal overlap between sound–target processing and the reactivation of an auditory component by the visual prime (for similar consideration, see Riou et al., 2014). It is therefore essentially this variability in the influence of the prime as a function of SOA that shows the perceptual nature of the cross-modal learnt unit. In sum, with our paradigm, we are able to test the implication of a cross-modal integration mechanism during learning.

In Experiment 2a, we showed that learning a congruent cross-modal stimulus produces a priming effect consistent with previous findings (i.e., interference followed by facilitation depending with increasing SOA, see Brunel et al., 2009). This confirms that participants exposed to an association between a visual component and an auditory component presented simultaneously created an integrated memory trace (see Versace et al., 2014) or event (see Zmigrod and Hommel, 2013). Once integrated, each component is no longer accessible individually without an effect of the other component. As a consequence, when participants see the visual component by itself, the auditory component is automatically activated as well. Moreover, the facilitation or interference of the priming effect was dependent on the temporal overlap between sound-target processing and auditory component reactivation (i.e., SOA manipulation). We interpreted this modulation as evidence of the perceptual nature of the memory component reactivated by the visual prime and thus the integration between these two components within a memory trace or event (see also, Brunel et al., 2009; Zmigrod and Hommel, 2010, 2013). However, these results are not just a replication of previous results because of our manipulation of the pre-experimental correspondence between sensory dimensions. Indeed, to the best of our knowledge this is the first time it has been shown that participants integrate the specific relation between perceptual features. In our previous studies (Brunel et al., 2009, 2010, 2013), the relation between the prime and the target was at a dimensional level (i.e., the prime and the target shared or did not share a sound dimension). With Experiment 2a, the prime-target relation is at the feature level (i.e., prime-target relations were congruent or incongruent with the previously learned associations). Moreover, we showed that participants actively learned such a relation despite the fact that the relation between the features is already congruent. This is evident by comparing the results observed in Experiment 1 and those observed in Experiment 2a. In Experiment 1, we showed a cross-modal congruency effect between visual lightness and auditory frequency. Participants were faster at processing congruent stimuli (i.e., either dark gray + low-pitched or light gray + high-pitched) compared to incongruent ones (i.e., the opposite mapping). In Experiment 2a, this effect was modulated by the SOA. According to our previous work (see Brunel et al., 2009, 2010, 2013), this modulation necessarily indicates that participants have learned a new perceptual unit. Otherwise, we should have only observed a replication of Experiment 1.

In Experiment 2b, we showed that, even when participants learn an incongruent association, the same pattern of priming effect is still observed at test. This result indicates that learning incongruent stimuli does not disrupt the cross-modal integration. However, a comparison of the priming effect observed between Experiments 2a,b indicates a smaller priming effect with learned incongruent units than with congruent ones. It is possible that the cross-modal correspondence influences integration. Indeed, the difference in the size of the priming effect between Experiments 2a,b might be due to different decay functions over the time. Because the priming effect reported in our experiments is a consequence of the newly integrated units (i.e., 32 presentations during learning), one might assume that the units will decline over time. In other words, since bimodal incongruent stimuli were only learned during our experiment, the association would probably be expected to decay faster than a congruent correspondence that has the strength of having been reinforced frequently in the past (see Marks, 1987). So our results seem to indicate that only a weak form of integration can be created in such a short period of time.

Spence (2011) proposed cross-modal correspondences can be understood in terms of Bayesian priors. The general idea is that humans may combine stimuli in a statistically optimal manner by combining prior knowledge and sensory information and weighting each of them by their relative reliabilities. In such an approach, cross-modal correspondences could be modeled in terms of prior knowledge (see Ernst, 2007; Parise and Spence, 2009; Spence, 2011). According to this model, the cognitive system establishes relations (or couplings) between stimuli in order to adapt to the situation and its constraints. The prior knowledge about the stimulus mapping has a consequence on the coupling of the stimuli (or the integration, see Ernst, 2007). The greater the prior knowledge in the system about the fact that two stimuli belong together, the stronger these stimuli will be coupled. In other words, the stronger the coupling, the most likely unisensory signals would be fused together, leading to the creation of a multisensory units. One major consequence would be an elevation of the discrimination threshold to detect internal conflict within a stimulus (e.g., asynchronous presentation, see Parise and Spence, 2009). In Experiment 1, we showed that prior knowledge about coupling between auditory frequency and visual lightness increases the perceptual processing of cross-modal congruent stimuli compared to incongruent ones. More interestingly, in Experiments 2a,b, we manipulated the prior knowledge distribution by creating an implicit novel association during the learning phase. This manipulation affected the cross-modal congruency effect that we observed in Experiment 1. The fact that we exposed participants to a pair of cross-modal features might have reduced the influence of coupling priors for the pair. As a consequence, the priming effect that we observed can be considered to be a measure of the modification of the influence of the coupling prior for the pair. As soon as one of element of the unit is presented the system makes an assumption about (or simulates) the presence of the other. Given that we observed a modulation of the priming effect depending on the SOA, we can argue that this assumption (or simulation) is more likely to occur at a perceptual stage rather than a decisional stage (for similar consideration, see Brunel et al., 2009, 2010, 2013; Evans and Treisman, 2010; Rey et al., 2014, 2015; Riou et al., 2014). Finally, it seems that learning congruent stimuli leads to the creation of “stronger” units (or coupling) over time because the system already has repeatedly experienced that these stimuli go together. Moreover, our results seem to indicate that the system does not need a large sampling of experiences to establish such prior knowledge distribution (or *coupling prior*). Indeed, the fact that we replicate our results in both Experiments 2a,b showed that the prior knowledge distribution depends on the experiences of the cognitive system rather than being exclusively built-in. Otherwise, we would not have observed a priming effect in Experiment 2b that conceptually replicated the one found in Experiment 2a.

## Conclusion

Our results support the idea that cross-modal correspondences, through the modification of coupling priors, modulate cross-modal integration during perceptual learning. Thus, perceptual consciousness could be considered as emerging from the integration of the current situation and the knowledge about prior situations. In that case, we can envisage that integration is crucial to conscious processing and might be a form of signature to those processing (see also, Dehaene et al., 2014)

However, there are still remaining open questions about how cross-modal integration might be linked to a very specific form of perceptual consciousness (e.g., synesthesia). Like for cross-modal correspondences, synesthetic experiences could be considered as structurally, semantically or statistically mediated (see Spence, 2011). However, recent findings seem to indicate that synesthetic experience could be understood as a consequence of some hyper-integration (or hyperbinding, see Mroczko-Wąsowicz and Werning, 2012) between an unusually large number of sensory or semantic attribute domains. This would be consistent with the idea that integration could be involved during the emergence of conscious states.

## Ethics statement

This research was conducted in accordance to the declaration of Helsinki, and had ethical approval from the Indiana University IRB office. All participants provided written informed consent and received partial course credit in return for their participation.

## Conflict of Interest Statement

The authors declare that the research was conducted in the absence of any commercial or financial relationships that could be construed as a potential conflict of interest.

## Footnotes

1 In speeded classification tasks participants have to discriminate one component of the stimulus as fast as possible while trying to ignore any other characteristics (see Marks, 2004).

2 In temporal order judgment task, participants have to make an unspeeded response on order relation in a trial sequence.

3 L used to refer as the perceived luminance of the eye whereas ^∗^a (green to red) and ^∗^b (blue to yellow) refer to the chroma.
